# Long-Term Feasibility and Outcomes of a Digital Health Program to Improve Liver Fat and Cardiometabolic Markers in Individuals With Nonalcoholic Fatty Liver Disease: Prospective Single-Arm Feasibility Study

**DOI:** 10.2196/72074

**Published:** 2025-09-12

**Authors:** Sigridur Björnsdottir, Hildigunnur Ulfsdottir, Elias Freyr Gudmundsson, Bartosz Dobies, Kolbrun Sveinsdottir, Ari Pall Isberg, Gudlaug E A Magnusdottir, Thrudur Gunnarsdottir, Tekla Karlsdottir, Gudlaug Bjornsdottir, Sigurdur Sigurdsson, Saemundur Oddsson, Vilmundur Gudnason

**Affiliations:** 1 Department of Molecular Medicine and Surgery, Karolinska University Hospital, Eugeniavägen 3, Solna, Stockholm, 171 77, Sweden, 354 8217107; 2 Heilsuklasinn, Reykjavik, Iceland; 3 Clinical Office, Sidekick Health, Vallakor 4, Kopavogur, 200, Iceland; 4 Doctoral School of Medical and Health Sciences, Faculty of Medicine, Jagiellonian University Medical College, Krakow, Poland; 5 Icelandic Heart Association, Kopavogur, Iceland; 6 School of Health Sciences, Faculty of Medicine, University of Iceland, Reykjavik, Iceland

**Keywords:** digital health program, nonalcoholic fatty liver disease, NAFLD, cardiometabolic health, digital therapeutics, liver, chronic, hepatic, cardiometabolic, cardiovascular, cardiology, weight, acceptability, digital health, metabolic syndrome, diabetic, diabetes, type 2 diabetes, BMI, lifestyle, exercise, physical activity, coaching, diet, dietary, nutrition, nutritional, patient education, coach, feasibility, fat, body composition, MAFLD, MASLD

## Abstract

**Background:**

A 12-week digital health program for nonalcoholic fatty liver disease (NAFLD) previously showed feasibility in engagement, program retention, and clinical outcomes. This study investigates whether improvements in cardiometabolic risk factors achieved during a 12-week active program were sustained over a subsequent 6-month follow-up period.

**Objective:**

The primary objective of this analysis was to evaluate whether the clinical improvements achieved after a 12-week program were maintained over the subsequent 6-month period, which did not include coaching or new intervention materials. In addition, the study aimed to assess participants’ retention and engagement with the maintenance program.

**Methods:**

In a 9-month, single-arm study using the Sidekick app (Sidekick Health), individuals with NAFLD and BMI >30 or metabolic syndrome or type 2 diabetes were included. The initial 12 weeks focused on providing education about diet, physical activity, stress management, and sleep, followed by 6 months without coaching or new intervention materials. The measured outcomes encompassed demographics, body composition, liver fat assessed using magnetic resonance imaging-proton density fat fraction (MRI-PDFF), and blood markers.

**Results:**

Of the 34 participants who completed the first 12 weeks, 28 (82%) completed the 9-month study measurements. The median age was 63.0 years (IQR 53.5‐71.0) and 57.1% (16/28) were women. At 9 months, compared to baseline, the mean weight loss was 4.0 kg (SD 5.0; *P*<.001). Liver fat decreased by 2.5% (SD 4.5; *P*<.001), with an 18.4% relative reduction. Systolic blood pressure decreased by 8.3 mm Hg (SD 13.4, *P*<.001) and diastolic by 2.5 mm Hg (SD 6.0; *P*=.02). Waist circumference decreased by 4.7 cm (SD 7.1; *P*<.001) and median glycated hemoglobin A1c (HbA_1c_) decreased by 19.5 mmol/mol (*P*<.001).

**Conclusion:**

Sustained improvements in liver fat and metabolic markers suggest that Sidekick Health’s digital program is a promising strategy for managing NAFLD without requiring continuous coaching.

## Introduction

As lifestyles have become more sedentary and diets have become hypercaloric, with high levels of refined sugars, grains, ultraprocessed foods, and sugar-containing beverages, nonalcoholic fatty liver disease (NAFLD) has emerged as a major public health concern. This rise parallels the global increase in obesity, type 2 diabetes, and metabolic syndrome [1,2]. NAFLD is now the most prevalent chronic liver disease globally, characterized by the accumulation of fat in the liver in the absence of excessive alcohol consumption or other identifiable secondary causes [3]. NAFLD encompasses a spectrum of liver conditions, from simple steatosis to its more severe form, nonalcoholic steatohepatitis (NASH), which can progress to fibrosis, cirrhosis, liver failure, and hepatocellular carcinoma [4]. NAFLD is a multisystem disease that is closely interlinked with metabolic syndrome, and they share common pathophysiological mechanisms and frequently coexist. NAFLD not only represents the hepatic manifestation of metabolic syndrome but may also contribute to its progression, establishing a mutual and bidirectional relationship between the 2 conditions [5,6]. In individuals with NAFLD, the leading cause of mortality is cardiovascular disease, followed by mortality from extrahepatic cancer, liver-related conditions (including hepatocellular carcinoma), and diabetes [7]. Furthermore, it is predicted that NAFLD and NASH will soon become the most common indication for liver transplantation, highlighting the growing socioeconomic burden associated with this disease [8].

Despite the rising prevalence of NAFLD, effective therapeutic options are still limited, with no approved pharmacological treatment currently available for its management [9]. Currently, lifestyle modifications focusing on dietary changes, increased physical activity, weight loss, and weight maintenance are the primary initial approaches for managing NAFLD [10]. However, the lack of standardized, evidence-based interventions and limited out-of-hospital monitoring often leads to suboptimal long-term results for individuals undergoing lifestyle interventions for NAFLD. This emphasizes the urgent need for innovative and scalable solutions to mitigate disease progression and improve long-term clinical outcomes. The use of digital technologies, such as smartphone apps, offers a promising approach to providing scalable and personalized interventions for individuals with NAFLD, with encouraging results reported in a recent study [11].

Sidekick Health is an Icelandic digital therapeutic company that has developed a digital health program (Sidekick-241 or SK-241) specifically designed for people with NAFLD and metabolic derangements. The emphasis of the SK-241 program is to improve nutritional status by focusing on limiting ultraprocessed foods, decreasing carbohydrates, increasing physical activity levels, reducing stress, and improving sleep. The results from the initial 12-week active phase of the SK-241 digital health program have been previously published, showing excellent retention, engagement, and satisfaction, alongside improvements in liver-specific and cardiometabolic health outcomes [12]. In this study, we present the 9-month results, including the 6-month maintenance period, highlighting the potential longer-term benefit of the SK-241 digital health program.

## Methods

### Trial Design

This open-label, single-arm, prospective study was conducted over 9 months, from June 2022 to April 2023 in Iceland. The study period included an active 12-week digital health program delivered through the Sidekick app, followed by an optional 6-month maintenance period. Clinical assessments, including screening and pre- and postprogram clinical assessments, were carried out at baseline, 12 weeks, and 9 months at The Icelandic Heart Association.

### Nomenclature

Recent updates in the nomenclature for fatty liver disease have introduced the term Metabolic Dysfunction-Associated Steatotic Liver Disease (MASLD) as a replacement for NAFLD [13]. While this new terminology has been endorsed by several major liver societies, it has not yet reached universal acceptance and has sparked some debate and confusion within the scientific community [14,15]. Given that the diagnostic criteria and study design for the present study were developed and registered under the established NAFLD framework, and to maintain consistency with previous literature, we have retained the use of the term NAFLD in this manuscript. This decision aligns with current guidance suggesting that researchers may continue to use the nomenclature most appropriate to their study context during this transitional period [16]. Future studies will adopt the updated consensus terminology as standardization across the field progresses.

### Participants and Screening for NAFLD

The screening process has previously been described [12]. Briefly, 38 individuals between 18‐80 years old, with at least 1 of the following risk factors: BMI >30, metabolic syndrome or type 2 diabetes mellitus, and confirmed liver steatosis >5% with a noninvasive ultrasonography-based controlled attenuation parameter (CAP) assessment through a FibroScan device were invited to participate after giving informed consent and fulfilling the inclusion and exclusion criteria [17].

### Digital Health Program

The active 12-week digital health program has previously been described [12]. To summarize, the primary focus of the 12-week-long program was to reduce participants’ daily dietary carbohydrate consumption and improve their overall nutrition quality in small, achievable, and sustainable steps (eg, reducing added sugars and processed foods, prioritizing protein, and increasing vegetable consumption). A secondary focus was to increase daily physical activity levels, improve sleep quality, and reduce stress. The program included short daily missions aimed at increasing knowledge about NAFLD and its contributing factors. The daily missions included watching short educational videos, reading brief informational content, and logging meals and beverages, which consisted of taking a photo of the meal, assessing on a sliding scale how healthy the meal was, and evaluating hunger and satiety before and after the meal. Other missions were regular practice of mindfulness and meditation, logging daily energy levels, stress, and sleep quality on a sliding scale from 0‐10. The 12-week active program also provided participants with in-app health coach support (a person, not artificial intelligence [AI]), who gave weekly feedback on food logs and other in-app activities and answered participants’ questions as needed. In addition, at the beginning of the 12-week program, participants had the opportunity to have a 30-minute video call interview with the health coach for a baseline assessment.

After completing the 12-week program, participants were given the option to retain access to the app for an additional 6 months. During this maintenance period, no new content was provided; however, participants could access previously received educational materials and continue using features such as a food-logging tool, in-app step counter, and other features. Active health coach support was not included during the 6-month maintenance period.

### Outcome Measures and Covariates

The primary objective of this analysis was to evaluate whether the clinical improvements achieved after a 12-week program were maintained over the subsequent 6-month period, which did not include coaching or new intervention materials. In addition, the study aimed to assess participants’ retention and engagement with the maintenance program.

Participants were assessed at baseline, 12-week, and 9-month for demographic data, anthropometric measures, medical history, medications, and adverse events. Liver fat content was measured and quantified at these time points using MRI-PDFF with a multiecho chemical shift-encoded gradient-echo sequence [18]. A new MRI machine was used for the 9-month follow-up visit. Analysis confirmed that the 2 machines produced comparable results, with an intraclass correlation coefficient of 0.97 (Multimedia Appendix 1). Body composition was assessed at baseline, 12 weeks, and 9 months, with a dual-energy x-ray absorptiometry (DXA) [19]. Blood pressure was measured using an automatic blood pressure monitor. Blood samples were drawn at the same time points to measure complete blood count, alanine aminotransferase (ALAT), aspartate aminotransferase (ASAT), glycated hemoglobin A1c (HbA_1c_), fasting glucose and insulin for Homeostatic Model Assessment of Insulin Resistance (HOMA-IR), total cholesterol, high-density lipoprotein-cholesterol (HDL-C), low-density lipoprotein-cholesterol (LDL-C), triglycerides, and high-sensitivity C-reactive protein (hs-CRP). The Fibrosis-4 Index, a noninvasive tool to estimate liver fibrosis risk and categorize outcomes in low-, medium-, and high-risk fibrosis groups, was calculated at baseline, 12 weeks, and 9 months [20,21].

Participants were given the following questionnaires via an electronic Patient Reported Outcome (PRO): Depression, Anxiety, and Stress Scale (DASS-21), health-related quality of life (EQ-5D-5L), and the Morisky Medication Adherence Scale (MMAS-8) [22-25].

For an exploratory engagement analysis, study participants were divided into 2 groups depending on how engaged they were with the digital health program to assess if clinical outcomes were associated with in-app use and engagement. For the 6-month maintenance period, clinical outcomes were compared between those participants who remained active during the maintenance period (defined as being active 18 or more weeks of the 24 weeks) and those who were less active.

For a supplementary exploratory behavior change analysis, participants were administered an in-app questionnaire at baseline and again at week 12, focusing on behavior change. To analyze the pre-post responses, the answers were categorized into 2 groups (Yes or No, Disagree or Agree, Rarely True, Often True) and the frequency of each category was reported. The analysis included those who had answered the questionnaire at both baseline and week 12. The McNemar chi-square test with continuity correction was applied to evaluate the significance of changes in response rates from baseline to week 12.

### Statistical Analysis

A formal sample size calculation was not performed as this was a feasibility study. It was considered sufficient to aim for 30‐40 participants to obtain information on practical aspects of recruitment, in-app engagement, retention, and rates of acceptance, which were the primary outcomes of the study.

Changes in clinical assessments and PROs between follow-up time points were calculated as the mean and SD for approximately normally distributed variables (normality was analyzed with the Shapiro-Wilk test) or as the median and IQR for variables that did not satisfy normality criteria. Categorical data were calculated as frequencies and percentages. To compare baseline and follow-up outcomes, paired *t* tests were computed for approximately normally distributed data. In case the normality assumption was not met, nonparametric tests were computed (Wilcoxon signed-rank tests). Unless otherwise specified, all statistical tests were performed at the 5% (2-sided) significance level. Statistical analysis was performed in Stata (StataCorp) and R (version 4.0.3; R Foundation for Statistical Computing). All enrolled participants were included in the full analysis set. Missing data were imputed using the baseline observation carried forward provided that the participant was enrolled in the study and at least 1 of 3 measurements (baseline or follow-up at 12 weeks or 9 months) was collected. Moreover, missing baseline measurements in waist circumference, hip circumference, and low-density lipoprotein cholesterol were imputed for 1 participant using the next observation carried backward. The complete case analysis set included participants who attended both the baseline visit and the 12-week follow-up visit.

### Ethical Considerations

The National Bioethics Committee of Iceland and the Data Protection Authority approved this study under the approval code 22‐075-Vl. All participants provided informed consent before enrolling in the study. All data were deidentified and analyzed in accordance with institutional protocols. Participants were given the option of seeking reimbursement for travel expenses not exceeding US $150 in total; no other compensation was provided. The study was registered at clinicaltrials.gov under the trial identifier NCT05426382.

## Results

### Participant Characteristics

A total of 28 participants completed the measurements following the maintenance program. The median age of those who completed was 63.0 years (IQR 53.5‐71.0), with 16 (57%) being female, and all participants identifying as Caucasian. None of the participants were smokers, and 42% held a university degree. At baseline, 89% of participants had obesity (BMI >30), 60% had type 2 diabetes mellitus, 75% had hypertension, 46% had hypercholesterolemia, and 40% had a history of cardiovascular disease. In total, 53% of participants reported taking antidiabetic medication, 85% antihypertensive medication, and 46% antilipidemic medication. During the 6-month maintenance period, 16 participants (42%) reported changes to their medication: 16 (42%) started new medications, 4 (10.5%) had dosage adjustments (in strength and or frequency), and 7 (18%) reported discontinuing medication. Of particular interest were changes that could influence metabolic outcomes. One participant started a glucagon-like peptide-1 agonist (GLP1-RA), semaglutide, along with metformin. Another participant switched from semaglutide to liraglutide, 1 increased their dose of semaglutide, and 1 participant stopped taking semaglutide. Other medication changes were considered irrelevant to the study outcomes by the study’s principal investigator.

### Retention and Engagement in the Maintenance Period

Of the 34 individuals who completed the 12-week active program, 28 (82%) individuals attended the third and final follow-up visit at 9 months. By the end of the 6-month maintenance period, 19 out of 38 participants (50%) were still retained in the app during the final week. In addition, 17 participants (45%) were active >5 days during 18 of the 24 weeks of the maintenance period. The median number of active days per week during this period was 2.38 (IQR 0.36‐6.08) days, and participants completed an average of 4.2 (SD 5.9) daily missions with the app per day.

### Metabolic Parameters

At month 9 compared to baseline (Table 1), participants in the full analysis set (n=38) demonstrated significant improvement in metabolic parameters.

The mean weight loss (SD) was 4.0 (5.0) kg (*P*<.001). The mean (SD) absolute reduction in liver fat was 2.5% (4.5) and the mean (SD) relative reduction was 18.4% (30.5) (*P*<.001). There was also a reduction in mean (SD) systolic blood pressure by 8.3 mm Hg (13.4) (*P*<.001) and in diastolic blood pressure by 2.5 mm Hg (6.0) (*P*=.02). The mean (SD) waist circumference decreased by 4.7 cm (7.1) (*P*<.001) and the median (IQR) HbA_1c_ was reduced by 18.5 mmol/mol (3-22) (*P*<.001).

When comparing the first 12 weeks to the subsequent 6 months maintenance period, all metabolic parameters remained significantly improved (Table 1), except for triglycerides (*P*=.15) and a slight increase in LDL-cholesterol. The mean (SD) LDL-cholesterol value was 2.9 mmol/L (1.1) at week 12 compared to 3.0 mmol/L (1.0) at month 9 (*P*=.04). In addition, diastolic blood pressure continued to decline during the maintenance period, resulting in a significant mean (SD) reduction of 2.5 mm Hg (6.0; *P*=.02) at month 9, which was not observed at week 12. These improvements were not explained by changes in medication or medication adherence (data not shown). Furthermore, the median (IQR) hs-CRP value significantly decreased from 3.0 mg/L (1.2‐5.2) at baseline to 2.4 mg/L (1.1‐3.9; *P*=.03) at month 9. In contrast, the reduction in hs-CRP at week 12 was not statistically significant. At baseline, the mean fasting s-insulin and HOMA-IR levels indicated insulin resistance. At week 12, we detected significant improvements in these variables and found that those improvements were sustained over the maintenance period (see Table 1). In addition, we saw further improvements in glycemic control during the maintenance period, with a median (IQR) reduction of 18.5 mmol/mol (3-22) (*P*<.001) in HbA_1c_ levels, compared with week 12. These data indicate that initial clinical improvements were sustained over the maintenance period.

There was a trend to an even better improvement in most of the metabolic parameters at 9 months compared to 12 weeks, although this was not significant.

The sustained improvements in weight loss and body composition were mirrored by sustained reduction in liver fat measured by MRI-PDFF. The mean (SD) liver fat percentage was 9.8% (6.6) at month 9 and 10.1% (6.5) at week 12. The mean (SD) relative reduction from baseline in MRI-PDFF liver fat value at month 9 was 18.4% (30.5), aligning closely with the reduction observed during the first 12 weeks of the study (*P*<.001).

**Table 1.** Differences in anthropometric, biochemical, and clinical measurements at baseline, week 12, and month 9 for the full analysis set.

Characteristics	Baseline (n=38)	12-week (n=38)	Month 9 (n=38)	Change from baseline to Week 12	Change from baseline to Month 9	Change from Week 12 to Month 9	*P* value baseline versus Week 12^a^	*P* value baseline versus Month 9^a^	*P value* Week 12 versus Month 9^a^
Anthropometry									
Weight (kg), mean (SD)	110.0 (18.5)	106.5 (18.4)	106.0 (19.4)	3.5 (3.7)	4.0 (5.0)	0.5 (4.4)	<.001	<.001	.48
Relative % change in weight*,* mean (SD)	N/A^b^	N/A^b^	N/A^b^	3.2 (3.4)	3.8 (4.9)	0.5 (0.4)	N/A^b^	N/A^b^	N/A^b^
BMI (kg/m2), mean (SD)	37.6 (5.8)	36.4 (5.8)	36.2 (6.1)	1.2 (1.3)	1.3 (1.7)	0.2 (1.4)	<.001	<.001	.46
Waist circumference (cm), mean (SD)	123.8 (12.2)	119.9 (12.2)	118.9 (13.7)	4.0 (5.1)	4.7 (7.1)	0.7 (5.5)	<.001	<.001	.45
Hip circumference (cm), mean (SD)	125.1 (14.0)	123.2 (13.3)	122.6 (14)	1.8 [0.0‐4.9]	2.5 (6.7)	0.6 (5.0)	.01	.03	.45
Waist to hip ratio, median, (IQR)	1.00 [0.95‐1.03]	0.99 [0.92‐1.03]	1 [0.9‐1.0]	0.00 [−0.01‐0.03]	0.02 (0.06)	0.0 [0.0‐0.0]	.09^d^	.02^d^	.33^d^
Liver assessment									
Liver fat MRI-PDFF (%), mean (SD)	12.3 (7.1)	10.1 (6.5)	9.8 (6.6)	2.2 (2.9)	2.5 (4.5)	0.3 (3.9)	<.001	<.001	.60
Liver fat MRI-PDFF mean relative change (%)^c^	N/A^b^	N/A^b^		19.4 (23.9)	18.4 (30.5)	5.8 (0.47)	N/A^b^	N/A^b^	N/A^b^
Liver stiffness measure (kPa), median (IQR)	6.4 [5.2‐9.6]	6.6 [5.3‐8.4]	6.4 [5.0‐8.4]	0.2 [−0.3‐1.6]	0.0 [−0.1‐1.2]	0.2 [−0.4‐1.8]	.11^d^	.11^d^	.15^d^
CAP score (dB/m), mean (SD)^e^	343.6 (34.8)	310.3 (47.2)	315.2 (54.3)	33.3 (39.7)	16.0 [0.0‐50.0]	−4.9 (51.6)	<.001	<.001	.56
Body composition									
Total body region fat, %, median (IQR)^f^	46.6 [39.4‐52.4]	44.3 [37.8‐52.2]	44.4 [8.1‐51.4]	0.9 (1.4)^c^	1.0 (2.6)	0.1 (2.4)	<.001	<.001	.81
Fat mass, kg ^f^	50.3 (13.8)	48.1 (14.5)	47.8 (14.5)	2.2 (2.7)	2.4 (3.9)	0.2 (3.7)	<.001	<.001	.72
Lean mass, kg^f^	56.3 (10.1)	55.6 (9.7)	55.3 (8.9)	0.7 (1.7)	1.0 (2.4)	0.3 (2.3)	.008	<.001	.42
Blood pressure (mm Hg), mean (SD)									
Systolic	141.4 (17.1)	135.4 (17.3)	133.1 (14.7)	6.0 (13.5)	8.3 (13.4)	2.3 (13.0)	.009	<.001	.29
Diastolic	83.6 (7.4)	82.5 (7.4)	81.1 (7.9)	1.2 (7.7)	2.5 (6.0)	1.3 (8.1)	.36	.02	.32
Biochemical measures									
HbA_1c_ (mmol/mol), median [IQR] ^g^	60.0 [56.0‐66.8]	60.0 [54.3‐64.0]	42.5 (39-55)	0.5 [−0.7‐3.8]	19.5 [2-22]	18.5 [3-22]	.03**^d^**	<.001**^d^**	**<.001^d^**
S-Glucose (mmol/L), median [IQR]^h^	6.2 [5.3‐7.4]	6.3 [5.4‐6.9]	6.3 [5.6‐6.8]	0.0 [−0.3‐0.4]	0 [−.2‐0.1]	0.0 [−0.4-.4.0]	.64^d^	.76^d^	.94^d^
S-Insulin (µU/ml), median [IQR]^i^	21.1 [16.4‐27.9]	19.0 [13.0‐25.0]	18.9 [13.5‐24.5]	3.2 [0.0‐5.4]	1.7 [0‐6.5]	0.0 [-3.4‐3.1]	.003**^d^**	<.001**^d^**	.64^d^
HOMA-IR (mmol/L), median (IQR)^j^	5.8 [4.3‐8.4]	4.8 [3.6‐7.2]	5.3 [3.9‐6.6]	0.4 [−0.2‐2.1]	0.4 [0.0‐2.0]	0.1 [−0.7‐0.9]	.02**^d^**	.007^d^	.68^d^
Total cholesterol (mmol/L), mean (SD) or median [IQR]	4.9 (1.3)	4.8 (1.2)	5.0 (1.2)	0.0 [−0.2‐0.2]	0.0 [−0.2‐0.0]	0.1 [−0.5‐0.2]	>0.99^d^	.05^d^	.28^d^
LDL-C (mmol/L), mean (SD) or median [IQR]^k^	2.9 (1.1)	2.9 (1.1)	3.0 (1.0)	−0.1 [−0.3‐0.1]	0.0 [−0.2‐0.0]	0.0 [−0.3‐0.3]	.18^d^	.04^d^	.48^d^
HDL-C (mmol/L), mean (SD) or median [IQR]^l^	1.11 (0.23)	1.12 (0.19)	1.1 (0.2)	−0.01 (0.12)	0.0 [−0.1‐0.0]	0.0 [−0.1‐0.1]	.56	.27	.55
Triglycerides (mmol/L), median (IQR)	1.88 [1.35‐2.45]	1.68 [1.21‐1.90]	1.8 [1.1‐2.2]	0.14 [0.00‐0.47]	0.0 [0.0‐4.0]	0.0 [−0.4‐0.1]	**.**003**^d^**	.15^d^	.25^d^
hs-CRP (mg/L), median [IQR]^m^	3.0 [1.2‐5.2]	2.5 [1.1‐3.9]	2.4 [1.1‐3.9]	0.1 [-0.1‐0.7]	0.0 [0.0‐0.8]	0.1 [−0.3‐1.0]	.14^d^	.03^d^	.27^d^
ALAT (IU/L), median [IQR]^n^	21.4 [18.2‐30.2]	23.2 [18.4‐32.0]	22.1 (18.7‐29.1)	0.0 [−6.8‐2.8]	0.0 [−5.0‐2.9]	0.8 [-1.6‐7.0]	.37^d^	.80^d^	.18^d^
ASAT, (IU/L), median (IQR)^o^	20.8 [17.9‐24.8]	22.3 [18.0‐25.5]	19.4 (16.7‐23.2)	0.4 [−2.5‐2.5]	0.0 [−0.2‐3.8]	0.8 [−1.2‐4.1]	.53^d^	.16^d^	.22^d^
Fibrosis-4 Index, median (IQR)^p^	1.08 [0.78‐1.34]	1.08 [0.75‐1.21]	1.0 (0.7‐1.2)	0.01 [−0.06‐0.07]	0.0 [0.0‐0.2]	0.8 [−1.2‐4.1]	.58^d^	.02^d^	.**03^d^**

a Paired *t* tests were computed for approximately normally distributed data.

b N/A: not applicable.

c MRI-PDFF: magnetic resonance imaging proton density fat fraction.

d For nonnormal data, nonparametric tests were computed (Wilcoxon signed-rank tests).

e CAP: controlled attenuation parameter.

f Measured by dual-energy ray absorptiometry.

g HbA_1c_: glycated hemoglobin A1c.

h s-glucose: Serum glucose.

i s-insulin: Serum insulin.

j HOMA-IR: homeostatic model assessment of insulin resistance.

k LDL-C: low-density lipoprotein cholesterol.

l HDL-C: high-density lipoprotein cholesterol.

m hs-CRP: high-sensitivity C-reactive protein.

n ALAT: alanine aminotransferase.

o ASAT: aspartate aminotransferase.

p Fibrosis-4: index for liver fibrosis.

At month 9, 29 out of 38 participants (76%) were classified as low risk of liver fibrosis based on the Fibrosis-4 Index, compared to 27 out of 39 (71%) at baseline. Six participants (16%) were classified as intermediate risk at month 9 compared to 7 (18%) at baseline, while 3 (8%) were classified as high risk at month 9 compared to 4 (11%) at baseline. Furthermore, the median Fibrosis-4 Index score significantly decreased over the 9-month study period, from 1.08 (IQR 0.78‐1.34) at baseline to 1.0 (IQR 0.7‐1.2) (*P*=.02).

No significant changes were observed in health-related quality of life (HRQoL; EQ-5D-5L), mental health (DASS-21), or medication adherence (MMAS-8) during the maintenance period, nor over the total study period (data not shown). In addition, the improvements in daily step count observed during the active 12-week program period were not sustained during the maintenance period, as recorded by the in-app step counter (data not shown).

### Associations Between App Engagement and Clinical Outcomes

An exploratory analysis was performed to assess the relationship between participants’ in-app activity during the maintenance period and their clinical outcomes. Previously, it was reported that participants who were highly engaged with the app during the active 12-week period (defined as visiting the app at least 5 days per week) experienced greater weight loss and liver fat reduction compared to less engaged participants [12]. A similar pattern was observed during the maintenance period, with highly engaged participants having significantly greater weight loss and relative liver fat reduction than those with lower engagement levels (see Table S1 in Multimedia Appendix 2).

### Behavior Change

In an exploratory analysis, participants’ self-reported dietary, exercise, and mental resilience behaviors were evaluated. Based on in-app questionnaires administered at Week 1 and Week 12, statistically significant improvements in healthy behaviors were found over the active period (see Table S2 in Multimedia Appendix 2).

### Adverse Events

In total, 26 adverse events were reported in the 6-month maintenance period (see Table S2 in Multimedia Appendix 2). No adverse events were considered related to the digital program as assessed by the investigator.

## Discussion

### Principal Findings

This study indicates that improvements in markers of cardiometabolic and liver-specific health markers, achieved during a 12-week active digital health program, can be maintained at 9 months, even without active coaching or new content being delivered during the 6-month maintenance period. We observed sustained weight loss, improvements in body composition, and reduction in liver fat, blood pressure, and glycemic control, all known as important key risk factors for cardiovascular disease [26]. At 9 months, we observed a significant reduction in hs-CRP levels and waist-to-hip ratio, which was not present at 12 weeks. Both markers are indicators of cardiovascular disease risk. Elevated hs-CRP reflects low-grade systemic inflammation that plays a key role in the development of atherosclerosis, while an increase in waist-to-hip ratio is an indirect measure of abdominal obesity, another well-established cardiovascular disease (CVD) risk factor [27,28]. In addition, at 9 months, we observed significantly lower median Fibrosis-4 Index values compared to baseline. The Fibrosis-4 Index is a biomarker of liver fibrosis and can potentially be used as a noninvasive alternative to liver biopsy for diagnosis and managing liver disease [21]. Our 9-month data showed that more individuals were categorized in the low-risk fibrosis group and fewer individuals were categorized in the intermediate-risk and high-risk groups at 9 months compared to baseline, suggesting improvements in liver health over the 9-month study.

The improvements observed in this study are not only clinically significant but also have important public health implications. NAFLD, with its potential progression to more severe forms of liver disease, poses a substantial and increasing burden on health care systems globally as its prevalence continues to rise [29,30]. The positive and sustained health outcomes shown here suggest that scalable digital programs, such as SK-241, have the potential to alleviate this burden by providing effective and accessible solutions. This program can reduce the strain on health care professionals and ease the overall pressure on health care systems.

Previous studies on lifestyle and behavior change interventions among individuals with NAFLD demonstrate, in general, a low success rate in achieving long-term weight management, a high dropout rate, and poor adherence to the prescribed interventions [31-33]. Moreover, the evidence on the effectiveness of digital health interventions for NAFLD is limited. To the authors’ knowledge, no previous studies have investigated the longer-term effectiveness of digital health programs for managing NAFLD [34]. Our study demonstrated that the improvements in weight loss, body composition, liver fat reduction, blood pressure control, insulin sensitivity, and glycemic control observed during the initial 12 weeks were sustained at 9 months. Suggesting that the program may induce potentially longer-lasting metabolic benefits. The digital nature of this program likely plays a role in fostering sustained engagement and support, helping participants adopt lasting behavior changes and effectively address the underlying causes of metabolic disturbances. Indeed, our exploratory analysis revealed that indicators of healthy behaviors, including diet, exercise, and mental resilience, were improved over the course of a 12-week program. These findings are consistent with the growing body of literature emphasizing the critical role of lifestyle modifications in mitigating metabolic dysfunction associated with NAFLD [35,36]. They contribute to the evolving narrative on the potential of digital health programs to serve as a scalable and accessible approach for improving cardiometabolic health in individuals with NAFLD and CVD in general.

Glucagon-like peptide-1 receptor agonists (GLP1-RAs) are considered promising treatment candidates for NAFLD and NASH and are indicated for the treatment of type 2 diabetes and obesity [37]. They have been shown to improve glycemic control and reduce weight, insulin resistance, and liver fat content [38]. A recent meta-analysis examining the effects of GLP1-RAs in individuals with NAFLD reported a mean weight loss of around 4 kg, comparable to the findings in our study. However, the mean relative reduction in liver fat content was greater, at 32% compared to the reduction observed in our study [38]. More recent studies have shown even higher weight loss and liver fat reductions after treatment with GLP1-RAs in individuals with NAFLD, making these medications a very promising treatment option for NAFLD [39]. Although these new medications have demonstrated strong clinical outcomes, there is potential for digital health solutions like SK-241 to complement GLP1-RAs treatments, provide additional support to patients using GLP1-RAs therapies, or serve as an alternative for those who cannot tolerate pharmacological treatment. However, this warrants further investigation.

The relatively high engagement and retention outcomes indicate that the 6-month maintenance period was well accepted by participants. However, we cannot assume that it was only or mainly the in-app activity during the maintenance period that drove the sustained health outcomes. Some may have continued to deploy the knowledge and behavior change tools they acquired in the first 12-week active program. Nevertheless, ongoing access to the app may serve as an important motivational tool for some participants, offering additional support to maintain health improvements and reinforce newly established habits. This suggests that the SK-241 program is a promising and valuable option in the comprehensive management of individuals with NAFLD and cardiometabolic conditions. However, further research is needed to determine the optimal duration of such a program to achieve sustained long-term benefits.

### Strengths and Limitations

A strength of this study was the high participant engagement and retention throughout the study period, enhancing the reliability of the longitudinal data. Furthermore, cardiovascular risk factors and liver fat content were assessed using objective and validated measures, with liver fat quantified using MRI-PDFF, a highly precise and reproducible imaging technique. Importantly, the observed reductions in liver fat content were accompanied by beneficial changes in cardiovascular risk markers. This temporal association supports the hypothesis that targeting liver fat may represent a feasible strategy to reduce cardiovascular risk, while also contributing to the ongoing scientific debate regarding the causal role of liver fat in cardiovascular disease [40,41].

Despite the encouraging findings, several limitations must be acknowledged. This was a preliminary, proof of concept study with a limited sample size of 28 participants and conducted under industry sponsorship. This characterization accurately reflects the early phase and exploratory nature of our work, which aimed to assess the feasibility and preliminary signals of efficacy, rather than to draw definitive clinical conclusions. The study was not powered to detect statistically significant differences in clinical endpoints or support subgroup analyses, such as by sex, which can be an important confounder in NAFLD research [42]. The study’s generalizability may be influenced by the specific characteristics of the study population, for example, all being Caucasian and with a relatively high education level. A potential limitation of this study is the absence of intermediate data collection points between the 12-week and 9-month follow-up assessments. While results at 9 months demonstrated sustained effects from the 12-week intervention period, the lack of more frequent measurements prevents a precise determination of whether a temporary “washout effect” (ie, a transient decline in the intervention’s benefits) occurred during this interval. Future studies could benefit from more frequent data collection to track the trajectory of the intervention’s effects more closely over time. Furthermore, missing values were imputed using baseline observation carried forward, which is a conservative approach but does not account for the fact that some individuals may have had different outcomes than their baseline values if they had attended the measurements, thus incurring a potential unmeasured bias. Further research is needed to explore the applicability of these results to a more diverse population and setting. In addition, the study did not address the potential impact of the program on other clinical outcomes, in addition to liver-related or cardiovascular morbidity and mortality, warranting further investigation. Moreover, as this was a single-arm study, results should be interpreted with caution. A full randomized controlled trial is needed to establish causal effects, to identify differences in outcomes by sex, and to validate these findings.

### Conclusions

This study adds to the growing evidence supporting the potential efficacy and sustainability of digital health programs in the management of NAFLD. Over a 6-month maintenance period, participants were able to sustain significant improvements in markers of liver and cardiometabolic health, indicating that the digital health program may produce long-lasting change, beyond the active phase. By targeting the root causes of metabolic disturbances and promoting long-term adoption of healthier habits, the SK-241 digital health programs provide holistic support for individuals with NAFLD. Moreover, the scalability and accessibility of the SK-241 digital health program have the potential to reduce the burden on health care systems. By empowering individuals to take an active role in managing their health remotely, these programs can alleviate pressure on traditional health care infrastructure and resources.

In conclusion, the findings of this study support the integration of digital health programs into the clinical management of NAFLD. Further research and implementation efforts are warranted to enhance the effectiveness and accessibility of these interventions, ultimately improving patient outcomes and reducing the burden on health care systems.

## Supplementary material

10.2196/72074 Multimedia Appendix 1 Supplemental report for MRI machine comparison.

10.2196/72074 Multimedia Appendix 2 Supplementary tables for outcomes by in-app engagement, behavior change, and adverse events.

## Abbreviations

ALAT: alanine aminotransferase
ASAT: aspartate aminotransferase
CAP: controlled attenuation parameter
DASS-21: Depression, Anxiety and Stress Scale
DXA: dual-energy x-ray absorptiometry
EQ-5D-5L: health-related quality of life
GLP1-RA: glucagon-like peptide-1 agonist
HbA_1c_: glycated hemoglobin A1c
HDL-C: high-density lipoprotein-cholesterol
HOMA-IR: Homeostatic Model Assessment of Insulin Resistance
hs-CRP: high-sensitivity C-reactive protein
LDL-C: low-density lipoprotein-cholesterol
MASLD: Metabolic Dysfunction-Associated Steatotic Liver Disease
MMAS-8: Morisky Medication Adherence Scale
MRI-PDFF: magnetic resonance imaging-proton density fat fraction
NAFLD: nonalcoholic fatty liver disease
NASH: nonalcoholic steatohepatitis
PRO: Patient Reported Outcome
T2D: type 2 diabetes
