# Supplementary material for: Long-Term Feasibility and Outcomes of a Digital Health Program to Improve Liver Fat and Cardiometabolic Markers in Individuals With Nonalcoholic Fatty Liver Disease: Prospective Single-Arm Feasibility Study
Source: JMIR Cardio. 2025 Sep 12;9:e72074. doi: 10.2196/72074 (PMC12431163; doi:10.2196/72074)
Supplement: Multimedia Appendix 1 [file cardio-v9-e72074-s001.pdf]

1 Introduction

2 Aim

3 Materials and methods

4 Results

5 Conclusions

MRI Machine Comparison Report

1 Introduction

The Icelandic Heart Association renewed MRI machines between the 12 week study visit and the nine month study visit. A comparative investigation was conducted in order to assess the effects of these changes on liver fat comparisons between study visits.

2 Aim

This aim of this report is assess the variability and agreement in liver fat measurements obtained from repeated scans using an older MRI and a new MRI machine. This report analyzes the differences between two MRI machines in both intra-machine and inter-machine comparisons. The the assessment will conclude whether there are systematic measurement errors and provide potential correction factors if needed.

3 Materials and methods

The study design involved two series of repeated MRI scans on a cohort of 25 volunteers. Each volunteer underwent two scans with the same MRI device, with a brief interval where were they were required to stand up between scans. The percentage of liver fat was measured from MRI images using the MRQuantif method at an offline workstation.

3.1 Technical information

Liver fat was assessed using: MRI-PDFF with a multiecho chemical shift–encoded gradient-echo sequence

- *Older MRI (Machine 1)*: 1.5 T Signa Twinspeed EXCITE system (General Electric Medical Systems, Waukesha, WI), Starmap (2D multi echo gradient echo mfgre) sequence and MRQuantif software
- *Newer MRI (Machine 2)*: 1.5 T Signa Explorer AIR-DL (General Electric Medical Systems, Waukesha, WI), Starmap (2D multi echo gradient echo mfgre) sequence and MRQuantif software

3.2 Statistical analysis

- **Intra/inter:**
    - Bland-Altman plots
    - Scatter plots

    - Means, standard deviations, and coefficient of variation (CV)
    - Intra-class correlation coefficient (ICC) Type: agreement
- **Inter:**
    - Mixed effects models for the inter analysis to evaluate the effect of MRI machines, adjusted for:
        - *Fixed effect*: MRI machine, sex, weight, and time between measurements, *random effects*: subject id

---

4 Results

4.1 Baseline characteristics

Variable	Measure
Age	40.9 ± 14.1
Sex	Male: 9, Female: 16
Weight	78.2 ± 18.1
Days Between Inter Measurements	49 ± 22.4

4.2 Intra analysis

4.2.1 Machine 1

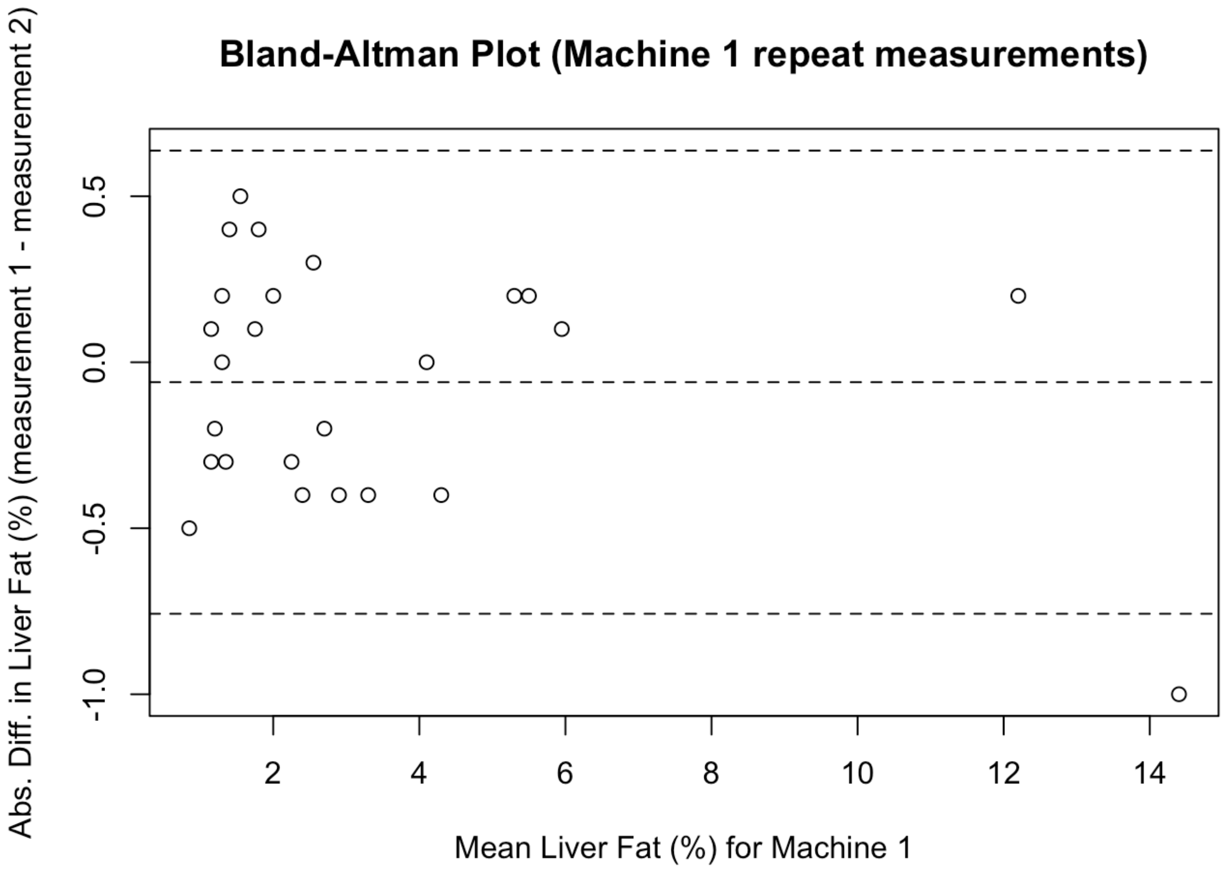

Figure 4.1: Bland-Altman plot comparing values from Machine 1

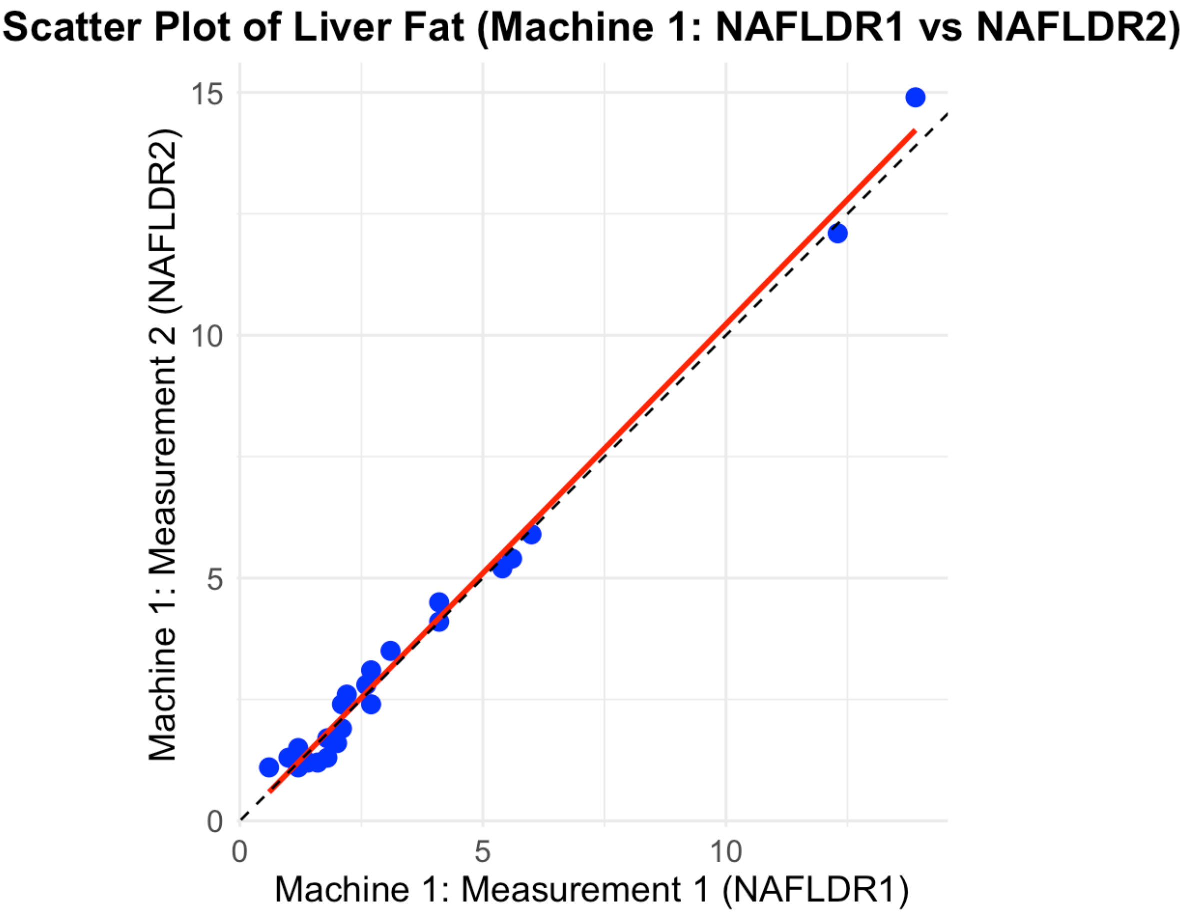

Figure 4.2: Scatter plot for Machine 1 repeated measurements. The dashed line is the 1:1 diagonal. The red line is a linear regression line without adjustments.

4.2.2 CV

Table 4.1: Summary of Liver Fat Measurements by MRI Machine

Measurement	Mean Liver Fat (%)	SD Liver Fat (%)	CV (%)
Measurement 1	3.4	3.3	98.0
Measurement 2	3.4	3.4	99.3

4.2.3 ICC

```
Single Score Intraclass Correlation

  Model: oneway
  Type : agreement

  Subjects = 25
    Raters = 2
    ICC(1) = 0.994

F-Test, H0: r0 = 0 ; H1: r0 > 0
  F(24,25) = 356 , p = 3.72e-26

95%-Confidence Interval for ICC Population Values:
 0.987 < ICC < 0.998
```

Table 4.2: Intraclass Correlation Coefficient (ICC) Results for MRI Machine #1 - two measurements

ICC_Value	Lower_CI	Upper_CI	P_Value
0.994	0.987	0.998	0

4.2.4 Machine 2

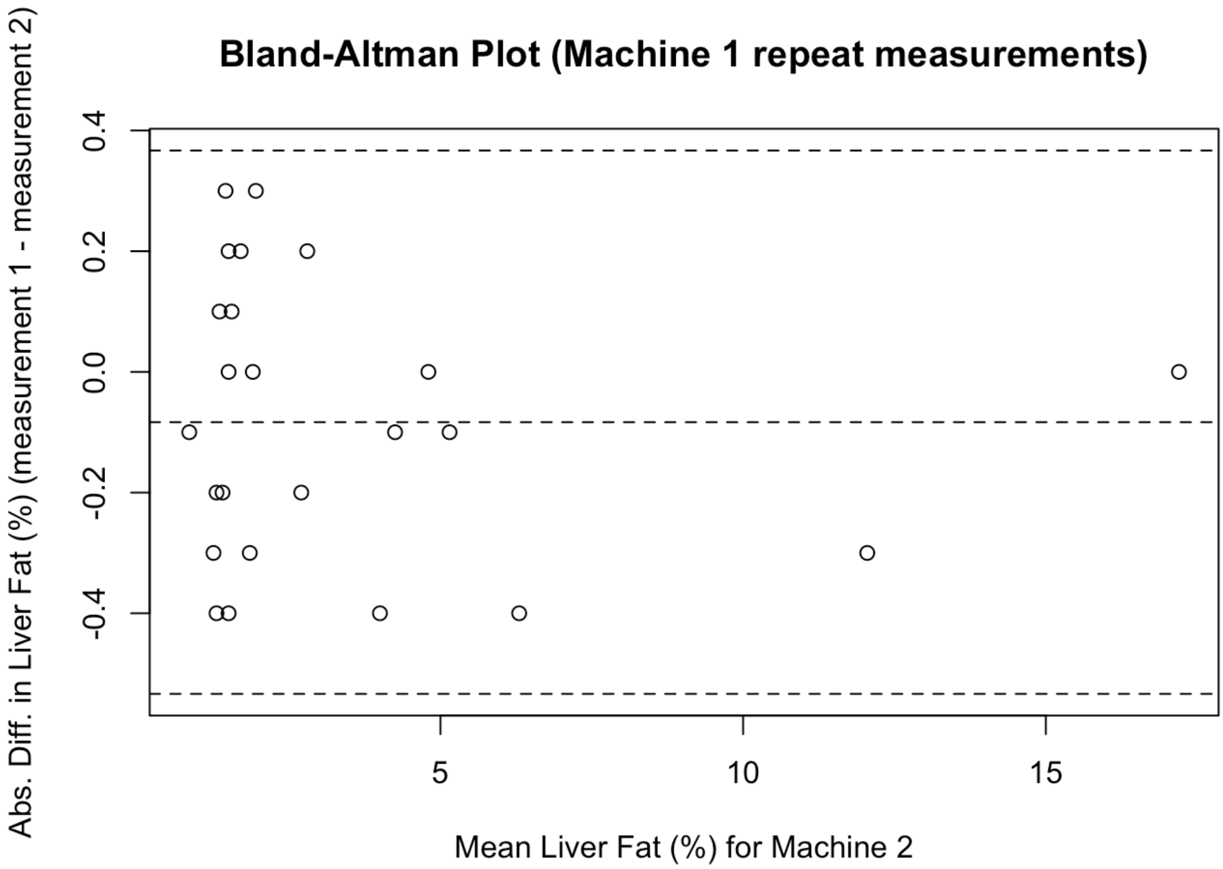

Figure 4.3: Bland-Altman plot comparing values from Machine 1

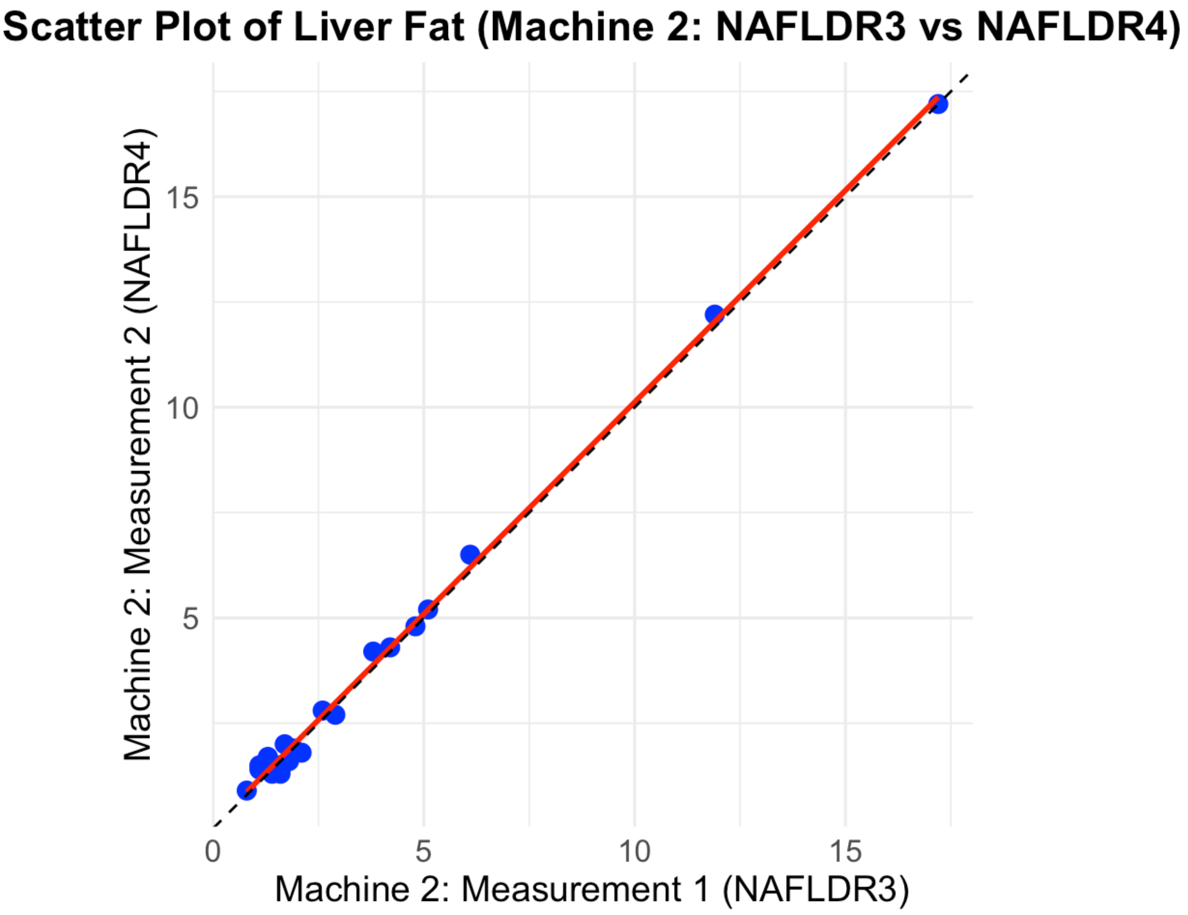

Figure 4.4: Scatter plot for Machine 2 repeated measurements. The dashed line is the 1:1 diagonal. The red line is a linear regression line without adjustments

4.2.5 Coefficient of variation

Table 4.3: Summary of Liver Fat Measurements by MRI Machine

Measurement	Mean Liver Fat (%)	SD Liver Fat (%)	CV (%)
Measurement 1	3.4	3.8	113.5
Measurement 2	3.4	3.8	111.6

4.2.6 ICC

```
Single Score Intraclass Correlation

  Model: oneway
  Type : agreement

  Subjects = 24
    Raters = 2
    ICC(1) = 0.998

F-Test, H0: r0 = 0 ; H1: r0 > 0
  F(23,24) = 1018 , p = 1.24e-30

95%-Confidence Interval for ICC Population Values:
 0.996 < ICC < 0.999
```

Table 4.4: Intraclass Correlation Coefficient (ICC) Results for MRI Machine #2 - two measurements

ICC_Value	Lower_CI	Upper_CI	P_Value
0.998	0.996	0.999	0

4.3 Inter analysis

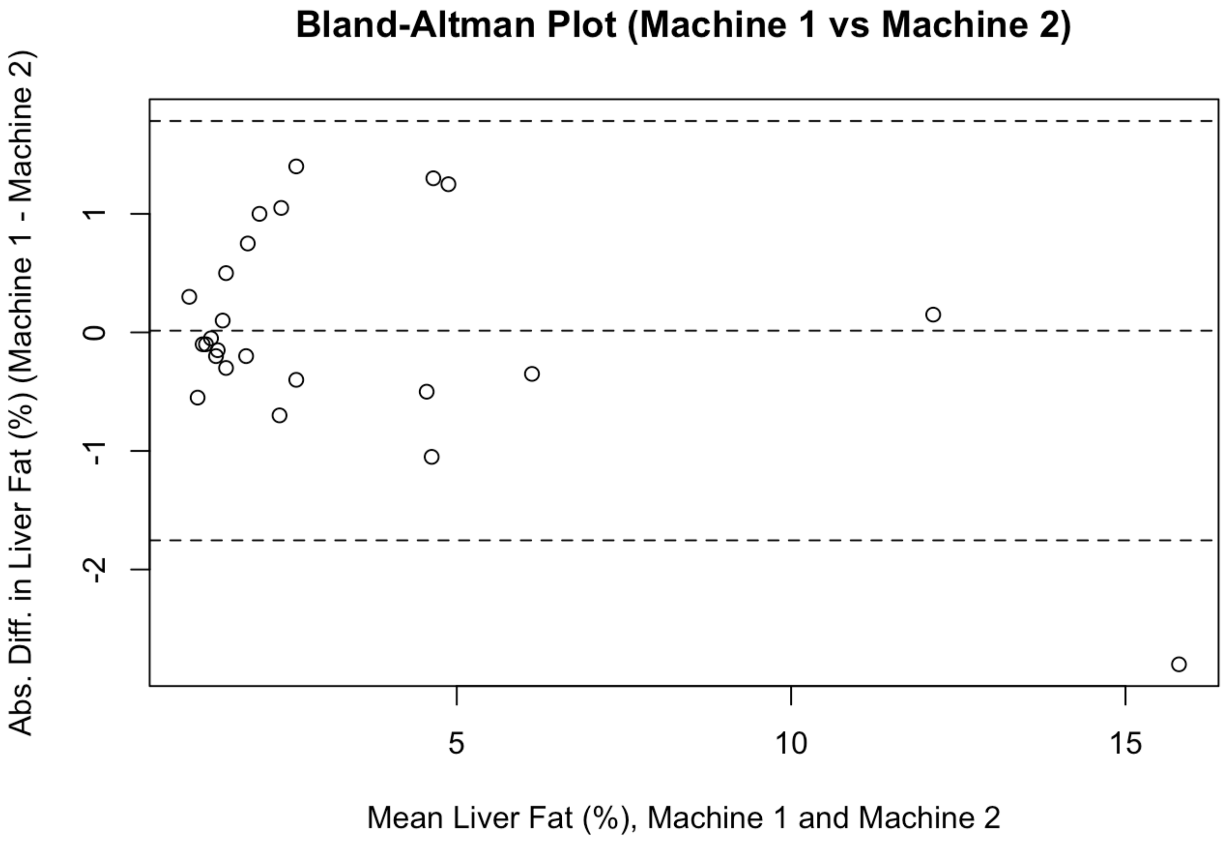

Figure 4.5: Bland-Altman plot comparing values from Machine 1 to Machine 2

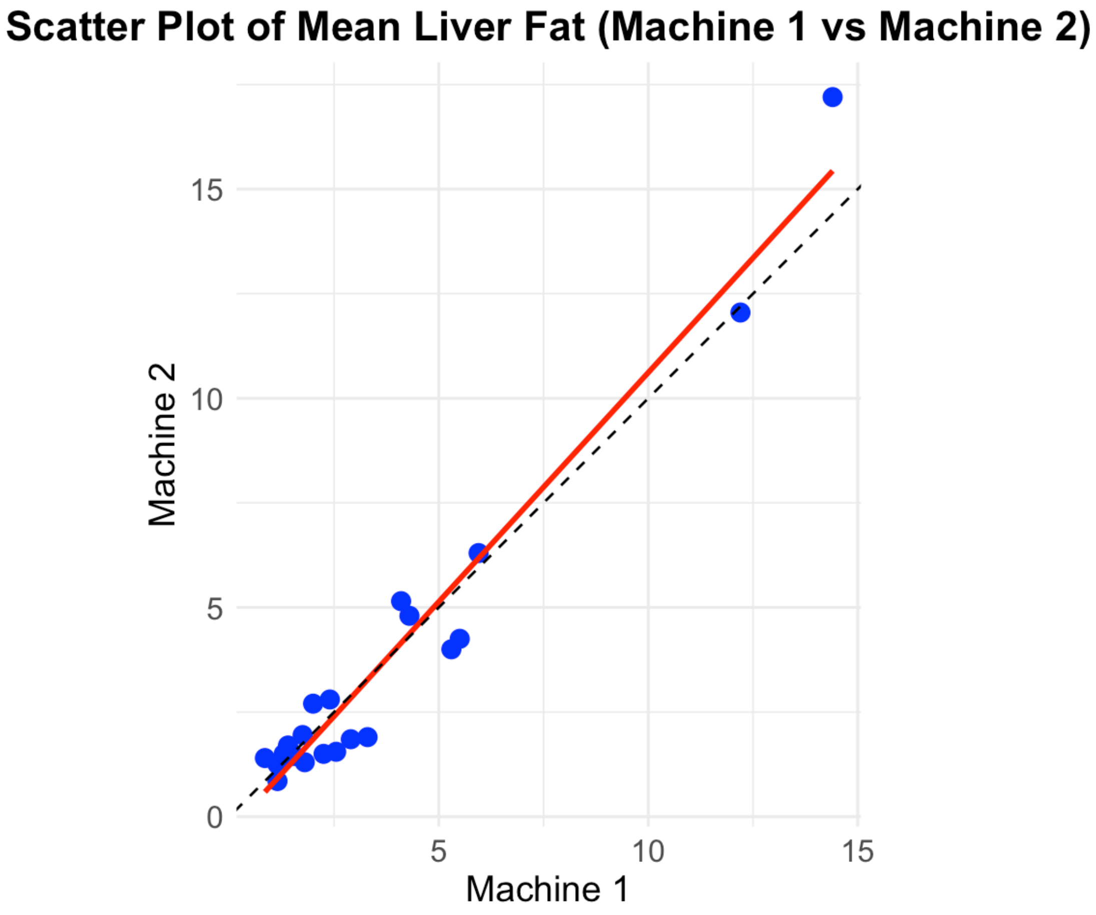

Figure 4.6: Scatter plot for individual measurements, machine 1 vs. machine 2. The dashed line is the 1:1 diagonal. The red line is a linear regression line without adjustments.

Overall, the Bland-Altman plot suggests that the two MRI machines have a high level of agreement in measuring liver fat, with minimal bias and acceptable levels of variability. The machines can be considered reliable for measuring liver fat with little systemic error.

4.3.1 t-test

```

	Paired t-test

data:  ba_data$m1_mean_liverff and ba_data$m2_mean_liverff
t = 0.079175, df = 23, p-value = 0.9376
alternative hypothesis: true mean difference is not equal to 0
95 percent confidence interval:
 -0.3664454  0.3956121
sample estimates:
mean difference 
     0.01458333 
```

4.3.2 CV

Table 4.5: Summary of Liver Fat Measurements by MRI Machine

Machine	Mean Liver Fat (%)	SD Liver Fat (%)	CV (%)
Machine 1	3.4	3.3	97.7
Machine 2	3.4	3.8	111.3

4.3.3 ICC

```
 Single Score Intraclass Correlation

   Model: oneway 
   Type : agreement 

   Subjects = 24 
     Raters = 2 
     ICC(1) = 0.97

 F-Test, H0: r0 = 0 ; H1: r0 > 0 
   F(23,24) = 66.2 , p = 1.59e-16 

 95%-Confidence Interval for ICC Population Values:
  0.933 < ICC < 0.987
```

Table 4.6: Intraclass Correlation Coefficient (ICC) Results for MRI Machines

ICC_Value	Lower_CI	Upper_CI	P_Value
0.97	0.933	0.987	0

4.3.4 Linear mixed-effects model

```
Linear mixed model fit by REML. t-tests use Satterthwaite's method [
lmerModLmerTest]
Formula: mean_liverff ~ machine + age + sex + weight + days_between +
    (1 | id)
   Data: data_mean

REML criterion at convergence: 194.7

Scaled residuals:
     Min       1Q   Median       3Q      Max
-2.04168 -0.29166  0.02267  0.23329  2.71390

Random effects:
 Groups   Name        Variance Std.Dev.
 id       (Intercept) 8.1528   2.8553
 Residual             0.4155   0.6446
Number of obs: 49, groups:  id, 25

Fixed effects:
                  Estimate Std. Error        df t value Pr(>|t|)
(Intercept)      -7.210553   5.984505 23.653026  -1.205   0.2402
machineMachine 2 -0.004397   0.186067 22.939124  -0.024   0.9814
age               0.020277   0.044158 20.222024   0.459   0.6510
sex              -0.021054   1.610916 22.092950  -0.013   0.9897
weight            0.108730   0.040100 26.405801   2.711   0.0116 *
days_between      0.026617   0.027578 20.264180   0.965   0.3458
---
Signif. codes:  0 '***' 0.001 '**' 0.01 '*' 0.05 '.' 0.1 ' ' 1

Correlation of Fixed Effects:
            (Intr) mchnM2 age    sex    weight
machinMchn2 -0.035
age         -0.415  0.009
sex         -0.865  0.018  0.225
weight      -0.768  0.025 -0.032  0.583
days_betwen -0.359 -0.012  0.138  0.225 -0.015
```

MRI Machine Comparison: There is no statistically significant difference between Machine 1 and Machine 2 for measuring liver fat,

4.3.5 Linear mixed-effects model - interaction

Check for possible interaction between weight and MRI machine.

```
Linear mixed model fit by REML. t-tests use Satterthwaite's method [
lmerModLmerTest]
Formula: mean_liverff ~ machine * weight + age + sex + days_between +
    (1 | id)
   Data: data_mean

REML criterion at convergence: 201.4

Scaled residuals:
     Min       1Q   Median       3Q      Max
-1.84072 -0.33836  0.01984  0.26068  2.51882

Random effects:
 Groups   Name        Variance Std.Dev.
 id       (Intercept) 8.1393   2.8529
 Residual             0.4245   0.6516
Number of obs: 49, groups:  id, 25

Fixed effects:
                         Estimate Std. Error        df t value Pr(>|t|)
(Intercept)             -7.088797   5.990104 23.738768  -1.183   0.2484
machineMachine 2        -0.607990   0.847316 21.983774  -0.718   0.4806
weight                   0.106472   0.040283 26.904886   2.643   0.0135 *
age                      0.019981   0.044137 20.247953   0.453   0.6556
sex                      0.014985   1.611448 22.054408   0.009   0.9927
days_between             0.026775   0.027565 20.291766   0.971   0.3428
machineMachine 2:weight  0.007692   0.010529 21.996755   0.731   0.4728
---
Signif. codes:  0 '***' 0.001 '**' 0.01 '*' 0.05 '.' 0.1 ' ' 1

Correlation of Fixed Effects:
            (Intr) mchnM2 weight age    sex    dys_bt
machinMchn2 -0.041
weight      -0.768  0.088
age         -0.415  0.011 -0.032
sex         -0.864 -0.022  0.579  0.224
days_betwen -0.358 -0.010 -0.016  0.138  0.225
mchnMchn2:w  0.034 -0.975 -0.084 -0.009  0.026  0.008
```

There was no significant interaction between weight and MRI machine.

5 Conclusions

- Both MRI machines provide highly consistent intra-machine and inter-machine liver fat measurements.
- Despite high variability within measurements as indicated by the CV, the excellent intra/inter agreement according to ICC, lack of significant differences in the paired t-test and mixed model, suggest that these machines are suitable for consistent liver fat assessments.
- The high CV does not necessarily impact the assessment of average values as the high ICC for agreement indicates that, on a group level, the average measurements are consistent between the machines.
- According the the mixed model weight is the primary driver of liver fat variation in this sample and both MRI machines yield comparable results.
- No systematic measurement errors were detected in visual and modelling inspections

- There is no need to adjust liver fat measurements for the two MRI machines.
