# Supplementary material for: Long-Term Feasibility and Outcomes of a Digital Health Program to Improve Liver Fat and Cardiometabolic Markers in Individuals With Nonalcoholic Fatty Liver Disease: Prospective Single-Arm Feasibility Study
Source: JMIR Cardio. 2025 Sep 12;9:e72074. doi: 10.2196/72074 (PMC12431163; doi:10.2196/72074)
Supplement: Multimedia Appendix 2 [file cardio-v9-e72074-s002.docx]

# Supplementary Materials

Table S1. Subgroup analysis of changes from week 12 (month 3) to month 9 for the clinical outcomes split by engagement status (active ≥ 5 days/week or <5 days/week). Analyzed for the full analysis set.

|  | Active ≥ 5 days/week in the maintenance period (n=12) | Active <5 days/week in the maintenance period (n=26) | Mean difference between groups | P value |
| --- | --- | --- | --- | --- |
| Weight loss, mean kg (SD) | -6.3 (4.6) | -3.0 (4.9) | -3.3 | **0.060** |
| Relative percentage change in weight, mean % (SD) | -6.4 (4.6) | -2.5 (4.6) | -3.9 | **0.020** |
| Absolute change in liver fat percentage, mean percentage points (SD) | -3.9(3.9) | -1.9 (4.4) | -2.1 | 0.199 |
| Relative percentage change in liver fat, mean % (SD) | -36.4 (29.7) | -10.1 (27.7) | -26.2 | **0.012** |

The questionnaire included the following items:

- To explore changes in dietary habits: “I regularly eat food I know isn’t good for me,” with responses on a Likert scale ranging from “Strongly disagree” to “Strongly Agree.” And “when everyone around me eats unhealthy food, I can easily resist the temptation,” with responses on a 5-point Likert scale from “Strongly disagree” to “Strongly agree.”
- To explore changes in physcial activity: “Do you participate in regular physical activity (at least 3-5 times per week)?” with response options: “I’m not interested in engaging in physical activity”, “No, but I would like to start within six months”,“No, but I would like to start within one month”,“Yes, I’ve been doing it for 1-6 months now”, “Yes, I’ve been doing it for more than six months now”
- To explore changes in resilience: “When uncomfortable feelings show up, I am able to notice them without acting on them, trying to control them, or take them away,” rated on a 5-point Likert scale from “Very rarely true” to “Very often true.”

Table S2. Participants’ responses to in-app questionnaire about their behaviors.

| **Statement** | **Chosen response** | **Baseline, n (%)** | **Week 12, n (%)** | ***P*-value^a^** |
| --- | --- | --- | --- | --- |
| I regularly eat food I know isn’t good for me | **Negative response** | **25 (83)** | **16 (53)** | **.04** |
|  | Agree | 10 (33) | 8 (27) |  |
|  | Strongly agree | 5 (17) | 1 (3) |  |
|  | Neither agree nor disagree | 10 (33) | 7 (23) |  |
|  | **Positive response** | **5 (17)** | **14 (47)** |  |
|  | Strongly disagree | 1 (3) | 6 (20) |  |
|  | Disagree | 4 (13) | 8 (27) |  |
| Do you participate in regular physical activity (at least 3-5 times per week)? | **Positive response** | **7 (29)** | **18 (75.0)** | **.001** |
|  | Yes, I’ve been doing it for 1-6 months now | 5 (21) | 10 (42) |  |
|  | Yes, I’ve been doing it for more than six months now | 2 (8) | 8 (33) |  |
|  | **Negative response** | **17 (71)** | **6 (25)** |  |
|  | I’m not interested in engaging in physical activity | 0 (0) | 0 (0) |  |
|  | No, but I would like to start within six months | 3 (12) | 2 (8) |  |
|  | No, but I would like to start within one month | 14 (58) | 4 (17) |  |
| When uncomfortable feelings show up, I am able to notice them without acting on them, trying to control them, or take them away. | **Positive** | **9 (30)** | **20 (67)** | **.003** |
|  | Often true | 6 (20) | 15 (50) |  |
|  | Very often true | 3 (10) | 5 (17) |  |
|  | **Negative** | **21 (70)** | **10 (33)** |  |
|  | Very rarely true | 2 (7) | 2 (7) |  |
|  | Rarely true | 5 (17) | 0 (0) |  |
|  | Sometimes true | 14 (47) | 8 (27) |  |
| When everyone around me eats unhealthy food, I can easily resist the temptation. | **Positive** | **14 (47)** | **20 (67)** | .11 |
|  | Agree | 11 (37) | 13 (43) |  |
|  | Strongly agree | 3 (10) | 7 (23) |  |
|  | **Negative** | **16 (53)** | **10 (33)** |  |
|  | Strongly disagree | 1 (3) | 1 (3) |  |
|  | Disagree | 5 (17) | 2 (7) |  |
|  | Neither agree nor disagree | 10 (33) | 7 (23) |  |

^a^ McNemar’s Chi-squared test with continuity correction to evaluate differences between the proportion of ‘negative’ and ‘positive’ responses at Baseline and Week 12.

Table S3. Occurrence of adverse events (AEs) during the 6-month maintenance period. Twenty-six adverse events were reported, four AEs were classified as serious*. However, no adverse events had a causal relationship to the digital health program, as assessed by the investigator.

|  |  |
| --- | --- |
| **AE description** | **Frequency reported, n** |
| Influenza | 8 |
| Pain in both shoulders | 3 |
| Covid-19 | 3 |
| Infection | 2 |
| Ingrown toenail | 2 |
| Hypothyroidism | 1 |
| Rosacea | 1 |
| Worsening back pain | 1 |
| Melanoma* | 1 |
| Urine incontinence* | 1 |
| Benign brain tumor* | 1 |
| Prostate cancer* | 1 |
| Common cold | 1 |
| *classified as serious adverse events |  |
